# Comparison of utility and organizational impact of reusable and single-use rhinolaryngoscopes in a tertiary otorhinolaryngology department

**DOI:** 10.3389/fsurg.2024.1380571

**Published:** 2024-10-07

**Authors:** Gunnhildur Gudnadottir, Louise Hafsten, Helena Dahl Travis, Kirsten Nielsen, Johan Hellgren

**Affiliations:** ^1^ Department of Otorhinolaryngology Head & Neck Surgery, Sahlgrenska University Hospital, Gothenburg, Sweden; ^2^ Institute of Clinical Sciences, Faculty of Medicine, Gothenburg University, Gothenburg, Sweden; ^3^ Department of Otolaryngology Head & Neck Surgery, Landspitali University Hospital, Reykjavik, Iceland; ^4^ Ambu A/S, Ballerup, Denmark

**Keywords:** endoscopy, nasopharyngoscopy, rhinolaryngoscopy, single-use, organizational impact

## Abstract

**Background:**

Flexible rhinolaryngoscopes are an important tool in otolaryngology. In recent years, single-use rhinolaryngoscopes (SURLs), which have been developed as an alternative to reusable scopes (RRLs), offer various advantages including less risk of contamination and elimination of the need for cleaning and reprocessing between procedures. This study aimed to compare procedure efficiency, organizational impact, and economic impact between SURLs and RRLs used for elective procedures conducted outside the otorhinolaryngology department in the hospital environment.

**Methods:**

In this randomized prospective study, either type of endoscope was tested by on-call otolaryngologists over a total of twelve weeks. The organizational impact was investigated using a quantitative research design. All categories of stakeholders responded to specific surveys based on profession; these included doctors (*n* = 13), those in managerial positions (*n* = 3), and other healthcare staff including technicians and nurses (*n* = 11). A micro-costing approach was used to evaluate resource utilization and cost of services. The trial was uploaded to clinicaltrials.gov (ID number: NCT0519821, https://clinicaltrials.gov/study/NCT05198219?intr=rhinolaryngo&rank=1).

**Results:**

Overall, 14 and 12 procedures were performed using the SURLs and RRLs, respectively. No significant differences were observed between the endoscopes in terms of procedure duration, reported image quality, or maneuverability. The SURLs were significantly superior in terms of four organizational impact parameters, namely, modes of cooperation and communication, vigilance and monitoring methods, working conditions and safety, and logistics. The estimated per-procedure cost of the RRLs was SEK 536 (€ 34,68).

**Conclusion:**

The cost and effectiveness of RRLs and SURLs is influenced by the healthcare setting, procedure volume, and duration of device use. The adoption of SURLs can improve safety, streamline processes, and potentially reduce the risk of disease transmission.

## 1 Introduction

Flexible endoscopes have revolutionized otorhinolaryngology (ORL) practice, as they allow direct visualization of the nose, throat, and airways in emergency, inpatient, or outpatient settings. These endoscopes are used for a wide variety of indications in ORL practice including airway obstruction, cancer surveillance, evaluation of swallowing, and assessment and treatment of the vocal cords. The image quality of flexible endoscopes has gradually improved owing to advances in technology from fiber optics to chip-on-tip systems with high definition 4 K cameras. Flexible rhinolaryngoscopy is conventionally performed using a reusable rhinolaryngoscope (RRL) which is cleaned and stored between procedures. Cleaning can involve manual reprocessing, sterilization, and automated endoscope reprocessing. Protective sheets can also be used to prevent contamination of the endoscope itself. In order to minimize the risk of damage and contamination between procedures, the endoscopes are often stored in cabinets with customized mounts. Damage induced by regular wear and tear and unfriendly handling can affect the camera chip, electrical wiring, or control wires that operate flexion of the distal end of the endoscope. In this context, a RRL in regular use has an expected life span of at least five years when handled with care.

In recent years, flexible single-use rhinolaryngoscopes (SURLs) have been developed as an alternative to RRLs for rhinolaryngoscopy. Ambu A/S developed a SURL, namely, the Ambu® aScope™ 4 RhinoLaryngo, in 2019. As the name suggests, the SURL is manufactured for use in a single patient for a single procedure; it is then recycled or destroyed. Several factors have led to the development of SURLs. Flexible endoscopy of the upper airway is performed in an environment that is colonized by various bacteria; invasive bacteria, viruses, and other contagious agents may additionally be found in the operative field. Studies have shown that contamination of flexible endoscopes may lead to the transfer of infections between patients (1). During the Coronavirus 2019 pandemic, flexible endoscopy of the upper airway was identified as a potentially highly contagious procedure (2). In situations such as these, the use of a sterile instrument, that may be unpacked directly after obtaining from the manufacturer and then used and disposed, could potentially minimize the risk of spread of viruses and bacteria (as compared to a RRL). It could also simplify ambulatory endoscopy procedures in hospital wards and emergency facilities, where handling of RRLs can be a challenge.

Image quality and endoscope handling are determining factors for the utility of specific endoscopes in clinical practice; economical and logistic factors also need to be considered. In addition, environmental issues related to the impact of their production, recycling, and repeated cleaning are pertinent factors that influence their use.

Although SURLs have been available for some years, and several single-use rhinolaryngoscopes have been introduced by various manufacturers, studies comparing the utility and organizational impact (OI) of SURLs and RRLs are lacking. Chateauvieux et al. compared single-use and reusable bronchoscopes in a large French university hospital; they found that the single-use bronchoscopes offered several advantages over reusable bronchoscopes in that setting. However, the higher costs associated with the use of the single-use bronchoscope were cited as a disadvantage (3). Unlike bronchoscopes, flexible rhinolaryngoscopes are typically designed without a working channel; this makes the cleaning procedure simpler, and may lead to differences in OI. This study compared the use of SURLs and RRLs among patients who were examined by a ORL doctor in hospitalized patients in inpatient wards. The aim was to compare efficiency, OI, and economic impact between RRLs and SURLs for elective procedures that were performed in inpatient wards.

## 2 Material and methods

This randomized prospective study compared flexible rhinolaryngoscopy procedures that were performed using RRLs and SURLs at the Department of Otorhinolaryngology, Head & Neck Surgery of the Sahlgrenska University Hospital, Gothenburg, Sweden, which is a tertiary referral center that serves a catchment area of 1.5 million inhabitants. The primary outcome measure was pre-procedure time from indication of the need for a rhinolaryngoscopy, as registered by the physician until the rhinolaryngoscope had been fully removed from the patient. Secondary outcome measures were procedure time, reprocessing time of RRL, user reported quality measures including image quality, maneuverability, ergonomics, and overall perception. Furthermore, secondary outcomes also included the cost per procedure of RRL, procedure completion rate, complications, and perceived organizational impact between RRL and SURL. All ambulatory consult procedures performed over 12 weeks, that fulfilled all inclusion criteria and none of the exclusion criteria, were considered eligible. The study was conducted in February, April and May of 2022.

The study was sponsored by the endoscope manufacturer, Ambu A/S, and the study protocol was designed and approved by both the investigators and the sponsor. The study was approved by the Swedish Ethical Review Authority (Dnr 2021-02769). The clinical investigation plan was formulated according to Council Directive 93/42 EEC of June 14, 1993 (amendment: 2007/47/EC); the guidelines of ISO 14155, ISO 14941, 21 CFR Part 812, and 50 (FDA); and the Helsinki Declaration. The trial protocol was registered on clinicaltrials.gov (ID number: NCT0519821).

### 2.1 Flexible endoscopes

The flexible endoscopes that were compared included a RRL, namely, C-Mac 8403 zx®, Karl Storz Endoscopes, Tuttlingen, Germany with a C-Mac portable monitor (also from 2015) and a SURL, namely, the Ambu® aScope™ 4 RhinoLaryngo Slim. Both types of endoscopes had been used regularly at the Department of ORL for some time before the start of the study.

Each scope was tested for a total of five weeks, (two weeks randomized had no eligible examinations). One type of endoscope (SURL or RRL) was used for one week at a time, and randomization was performed using the 365 RAND() function in Microsoft Excel. In addition to randomization, a separate investigator site file (one for each week) was maintained, in which the type of flexible rhinolaryngoscope (RRL or SURL) used for the specific study period was documented.

The investigating physicians were ORL specialists or registrars who were on-call during the daytime (7.30 am–5 pm) and answered emergency calls and referrals for consultation at the hospital (for patients who could not be transported to the ambulatory ORL unit. The investigators assessed and treated patients with ORL-related issues at hospital wards outside the ORL department. All doctors were trained in flexible endoscopic rhinolaryngoscopy and had all conducted at least five procedures of both SURL and RRL prior to recruitment in the study.

All included participants received oral and written information regarding the study and signed an informed consent form. The patients examined in this study were not associated with the study, and the decision to perform (or not perform) flexible rhinolaryngoscopy was made by the ORL doctor based on clinical assessment. The doctor also needed to be able to respond to the questionnaire and monitor time; emergency procedures (where efficiency and patient safety had to be prioritized) were not included in the study.

### 2.2 Time efficiency and utility

At each ambulatory examination, the doctor completed a report form, which included the time when the endoscope was picked up by the doctor at the Department of ORL's ambulatory unit and time at arrival at the patient bedside. The time points of initiation and conclusion of the examination, type of procedure (rhinoscopy, laryngoscopy, or assessment of swallowing ability), and category of medical issue (foreign body, trauma, infection, paresis (including neurological causes of dysphonia or dysphagia, cancer, or other) were also included. The report forms also included questions regarding image quality and the ergonomics of the flexible endoscope; responses were recorded on a 5-point Likert scale. If the examination had to be aborted for any reason, this had to be recorded on the form with the relevant reason. The full questionnaire has been provided in Supplementary Materials.

### 2.3 Organizational impact

After completion of all the procedures in this study, the OI was investigated using a quantitative research design. Specific surveys were completed by all categories of stakeholders based on profession; these included participating ORL doctors, managerial staff at the Department of ORL, and other healthcare staff including technicians and nurses who managed the flexible endoscopes at the Department of ORL. The survey was developed based on the defined parameters of OI for medical devices (3, 4); this allowed comparative evaluation of the impact of SURLs and RRLs on organizational aspects. Two parameters, namely, accessibility and architectural and infrastructural design, were omitted from the model owing to lack of relevance to the scope of the investigation.

The survey was completed on the online platform, SurveyExact. All questions in the survey were categorized in advance according to the parameters of OI. Respondents selected one of three options: favorable to SURLs, favorable to RRLs, or neutral (between SURLs and RRLs); results were reported as percentages. To minimize bias, the order of the questions was randomized by SurveyExact and the survey was constructed to ensure diverse vocabulary in its questions; both positive and negative framing were employed for advantages and disadvantages. The questionnaires were distributed by the primary investigator to ensure respondent anonymity in relation to financial support associated with the study (by Ambu A/S).

### 2.4 Cost assessment

**Figure 1:**
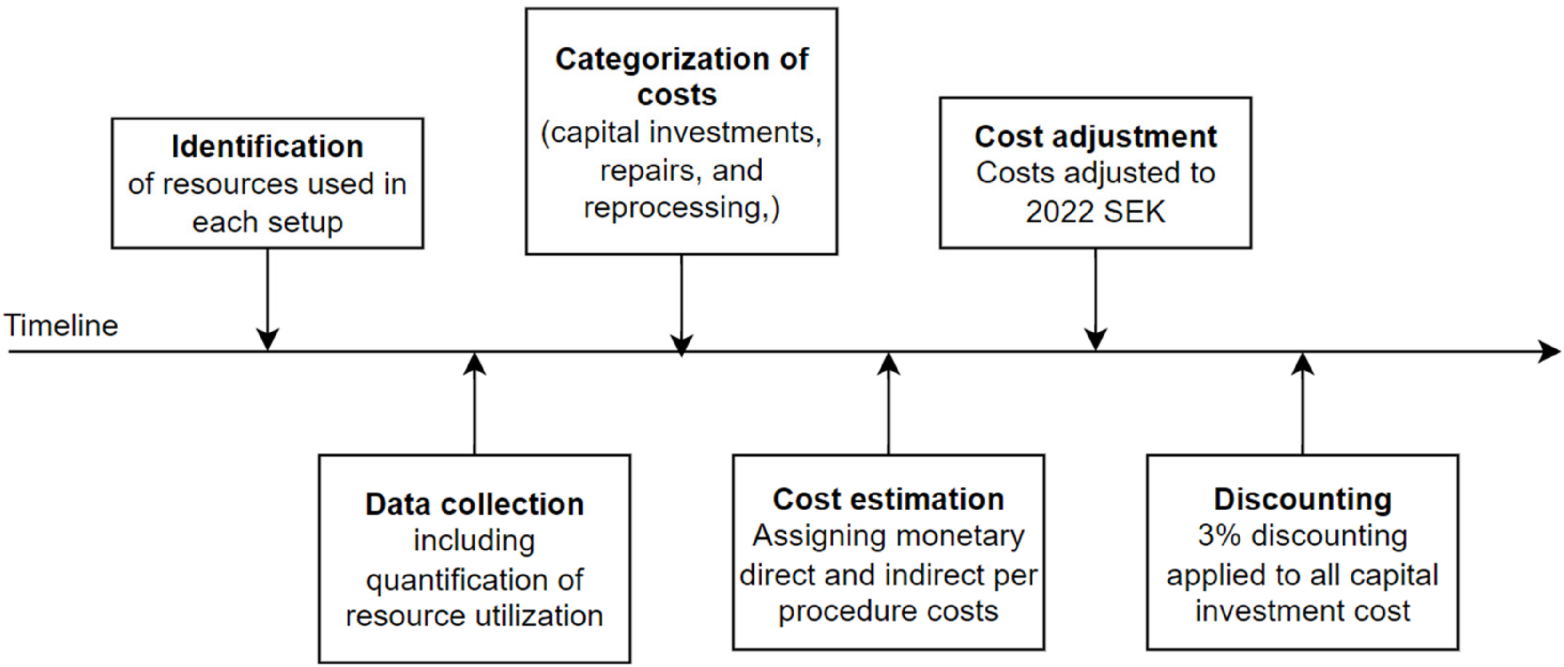
Cost analysis. Steps of the cost-analysis.

A micro-costing approach was used to evaluate resource utilization and cost of services in an ORL clinic. Trained data collectors prospectively measured the resources utilized during a procedure, including reprocessing and storage. Data pertaining to personnel time, utensils used, detergents used, repairs, and capital investment (e.g., automated endoscope reprocessing, monitors, and endoscopes) were recorded. Notably, the micro-costing approach involves the assigning of monetary values (2022 SEK) to each resource used in the clinic; the steps are demonstrated in Figure 1. All cost data related to RRLS were obtained at the study site, namely, the Sahlgrenska University Hospital, over a two-month period (May and June 2023). Information on additional associated costs was obtained from various sources, including the clinic administrative records and billing and accounting systems. These sources provided information on fixed costs, overhead expenses, and other financial data. Due to variability in SURL prices between countries and brands, the published costs were used in this study; this ensured more accurate capture of the comprehensive economic aspects of SURL costs.

### 2.5 Statistical analyses

Estimates showed that a minimum of 26 fully evaluable procedures (i.e., a total of at least 13 procedures with each of the 2 products) had to be included in the study to demonstrate the primary outcome with a power of 90%. Sample size calculation was based on a study on single-use vs. reusable bronchoscopes (5). All statistical analyses were done by an external statistician.

Descriptive statistics, including means, were computed to summarize resource utilization patterns and cost distributions. The Wilcoxon rank sum and Fischer's exact tests (two-sided) were used for comparison between groups. Sensitivity analyses were performed by varying key parameters to evaluate the robustness of cost estimates. In accordance with the reference case by Drummond et al., all RRL costs were annualized over a five-year period (6). Capital investments, such as those related to processors, light sources, and automated endoscope reprocessing, were annualized over an eight-year period. Furthermore, all capital costs were discounted by 3%, as per the reference case of the Institute for Clinical and Economic Reviews (7).

Data analysis was conducted using R Studio, and the relationship between the OI of RRLs and SURLs was evaluated using the chi-square test. A radar plot was constructed in accordance with the model described by Châteauvieux et al. (3), and the significance level for comparing the reusable and single-use setups was found for each OI parameter (after excluding all neutral answers). A *p*-value of <0.05 was considered significant for all analyses.

## 3 Results

Overall, 14 and 12 procedures were included using the SURLs and RRLs, respectively. Five cases were excluded after the ORL doctor-initiated data collection due to screen failure (three and two for SURLs and RRLs, respectively). During two of the designated weeks for the study, no eligible rhinolaryngoscopy procedures were available for inclusion, one week for SURL and one week for RRL, respectively. The questionnaires on OI were completed by the 13 ORL doctors included in the study. In addition, 11 healthcare staff (including technicians and nurses and others handling the flexible rhinolaryngoscopes at the Department of ORL) and three staff in managerial positions responded to the OI survey. The clinical experience (position) of the included doctors is shown in Table 1, and the indications for the flexible rhinolaryngoscopies stratified by endoscope type) are shown in Table 2.

**Table 1:** Physician experience.

Model	Registrar^a^	Specialist	Senior ORL consultant^b^	Total
Ambu	11 (79%)	2 (14%)	1 (7%)	14 (100%)
Reusable	8 (67%)	1 (8%)	3 (25%)	12 (100%)
Total	19 (73%)	3 (12%)	4 (15%)	26 (100%)
*P*-value	0.4354 Chi-squared test. 2-sided			

Experience of the physicians distributed by title.

^a^ Physician in specialisttraining.

^b^ Senior specialist (<5 years) and added responibilites (ORL, otorhinolaryngology).

**Table 2:** Indication for the rhinolaryngoscopy (multiple choices possible).

Group	Foreign body	Trauma	Infection	Cancer	Paresis^a^	Other^b^
Ambu	1 (7%)	0 (0%)	1 (7%)	2 (14%)	3 (21%)	10 (71%)
Reusable	1 (8%)	0 (0%)	0 (0%)	2 (17%)	0 (0%)	9 (75%)
*P*-value	1.0000	1.0000	1.0000	1.0000	0.2246	1.0000

Indication for performing the rhinolaryngoscopy.

^a^ Dysphonia and/or dysphagia, suspected neurological causes.

^b^ Including breathing problems, check of tracheostomy, and bleeding in the airway.

### 3.1 Time efficiency and utility

**Figure 2:**
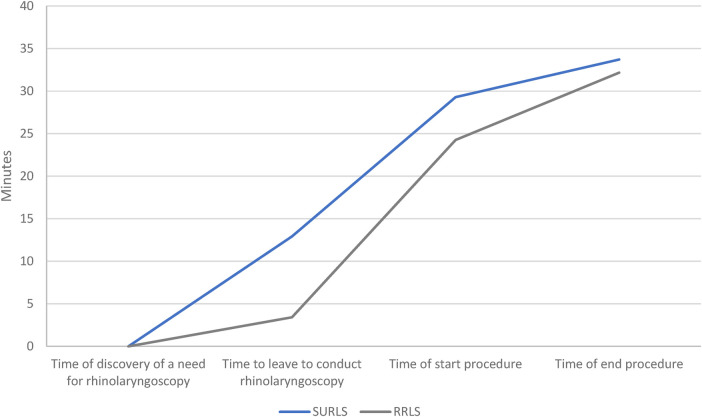
Time assessment. The curve (mean values) from indication for the need of rhino laryngoscopy (i.e., “Time point at which rhinolaryngoscopy was found to be indicated”) until the initial conclusion (i.e., “Time point at which procedure ended”).

**Figure 3:**
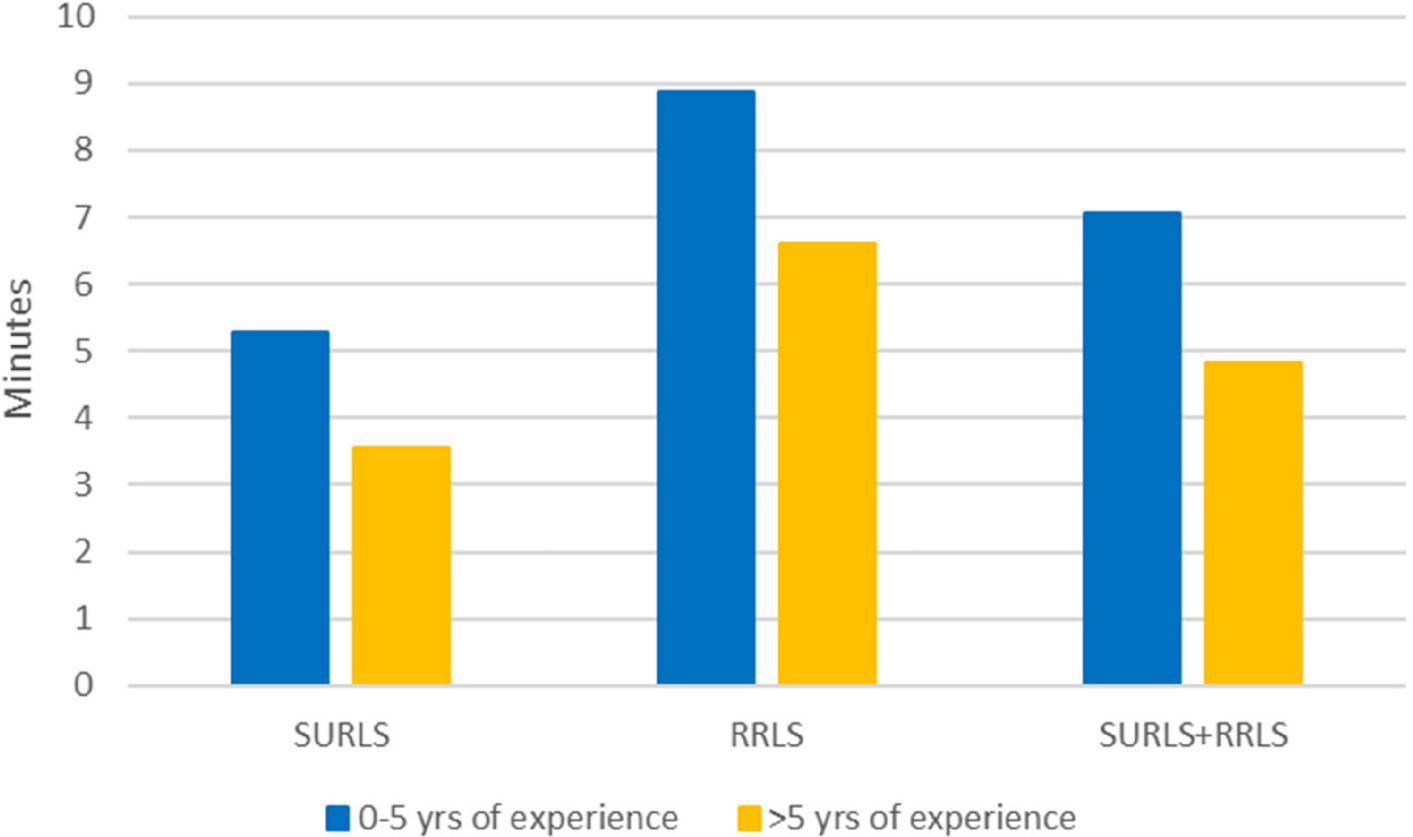
Mean time in minutes from start to end of procedure based on group and doctor experience (years). Mean time in minutes from “Start Procedure” to “End Procedure” by group and Doctors Experience (years).

For the RRLs, the average time spent on manual reprocessing was found to be 14 min per procedure. No significant difference was observed between the two endoscopes in terms of time elapsed between indication for a rhinolaryngoscopy and the point at which the rhinolaryngoscopy ended (Figure 2); no differences were found in terms of reported image quality (Table 3) and maneuverability of the endoscopes (Table 4). The mean time from the start to end of the procedure was shorter for SURLs (mean: 4.4 min, standard deviation: 3.0) than RRLs (mean: 7.9 min, standard deviation: 7.4); however, the difference was not statistically significant (Figure 3).

**Table 3:** Rating of image quality during the procedure.

Group	Category	Number of patients (%)
Ambu	Very poor	0 (0%)
	Poor	1 (7%)
	Acceptable	7 (50%)
	Good	1 (7%)
	Very good	5 (36%)
	Total	14 (100%)
Reusable	Very poor	0 (0%)
	Poor	1 (8%)
	Acceptable	6 (50%)
	Good	4 (33%)
	Very good	1 (8%)
	Total	12 (100%)
*P*-value Wilcoxon rank sum test (2–sided)		0.5549

Survey question: “How would you rate the image quality during the procedure?”.

**Table 4:** Rating maneuverability of the rhinolaryngoscope in the procedure performed.

Group	Category	Number of patients (%)
Ambu	Very difficult	1 (7%)
	Difficult	0 (0%)
	Acceptable	3 (21%)
	Easy	4 (29%)
	Very easy	6 (43%)
	Total	14 (100%)
Reusable	Very difficult	0 (0%)
	Difficult	0 (0%)
	Acceptable	2 (17%)
	Easy	5 (42%)
	Very easy	5 (42%)
	Total	12 (100%)
*P*-value Wilcoxon rank sum test (2-sided)		0.7867

Survey question: “How would you rate the maneuverability of the rhinolaryngoscope in the procedure performed?”.

### 3.2 Costs

**Figure 4:**
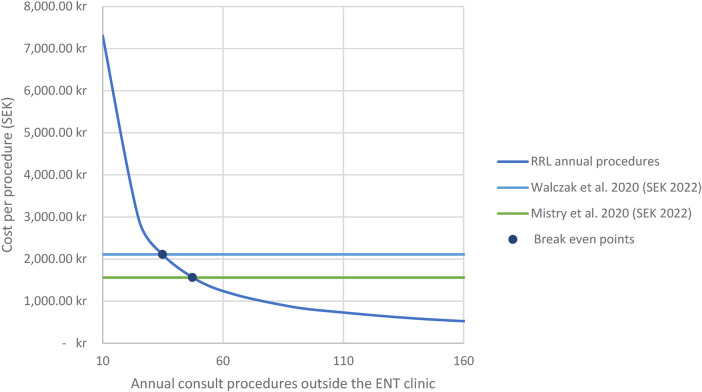
Cost scenario analysis. Scenario analysis of annual consult rhinolaryngoscope procedures outside the ENT clinic and cost per rhinolaryngoscope procedure. ENT, Ear, Nose and Throat; RRL, Reusable rhinolaryngoscope. All costs are in 2022 SEK (converted on 10.24.23). Based on micro-costing analysis, the total estimated per-procedure cost of RRL was SEK 536. The break-even points between SURLS and RRLS was found to be 47.2 and 34.9 procedures per year, respectively.

The estimated per procedure costs for RRLs was found to be SEK 536. This included per procedure costs for capital equipment (SEK 143), for repairs (SEK 314), and for reprocessing (SEK 79). The cost of RRLs is based on 156 annual rhinolaryngoscopies outside the ORL department. This number is derived from the study site and represents the average number of uses per year. Two instances of repairs were reported in the billing and accounting systems of the calendar year 2022 (SEK 48,000 and SEK 950, respectively) corresponding an average repair cost per procedure of SEK 314. Two published research papers were identified, presenting a per procedure cost of SURL and found per procedure cost of SEK 2,110.43 (8) and SEK 1,562.44 (9) (both converted to 2022 SEK). The breakdown per-procedure cost of RRLs is outlined in Table 5, and the per-procedure cost of SURLs is outlined in Table 6. The break-even point depends on the annual procedure volume and capital equipment cost and is found to be after 47.2 procedures or after 34.9 procedures per year, based on Mistry et al. (9) and Walczak et al. (8), respectively. Though the SURL setup also includes a monitor, which, similar to the RRL setup, declines over time, the upfront per-use cost for SURL is lower, since the SURL setup does not include any costs of reprocessing equipment and repairs. The per procedure costs for reusable and single-use endoscopy setup, and break-even costs are shown in Figure 4.

**Table 5:** Cost per procedure of reusable rhinolaryngoscopy.

Costing component	Cost per procedure (2022 SEK)
Capital equipment's	
- Reusable rhinolaryngoscope	91
- Portable monitor	38
- Automated endoscope reprocessor (AER)	14
Repairs	
- Endoscope repairs	314
Reprocessing	
- Personal protective equipment's	1
- Disinfections	1
- AER detergents	40
- Manual cleaning detergents	<1
Hands on time (salary)	
- Endoscope cleaning	<1
- Additional (e.g., repair shipment, ordering and filling of reprocessing equipment)	37
**Total**	**536**

Cost per procedure of reusable flexible rhinolaryngoscopy.

**Table 6:** Cost per procedure of single-use rhinolaryngoscopy.

	Cost per procedure (2022 SEK)	
Costing component	Mistry et al. 2020	Walczak et al. 2020
Capital equipment's		
- Rhinolaryngoscope		2106
- Portable monitor		4.5
- Automated endoscope reprocessor (AER)		NA
Repairs		
- Endoscope repairs		NA
Reprocessing		
- Personal protective equipment's		NA
- Disinfections		NA
- AER detergents		NA
- Manual cleaning detergents		NA
Hands on time (salary)		
- Endoscope cleaning		NA
- Additional (e.g., repair shipment, ordering and filling of reprocessing equipment)		NA
**Total**	**1,562** **^a^**	**2,110.5**

Cost per procedure of single-use flexible rhinolarngoscopy.

^a^ The costs are not itemized into separate components but are reported collectively per procedure.

### 3.3 Organizational impact

**Figure 5:**
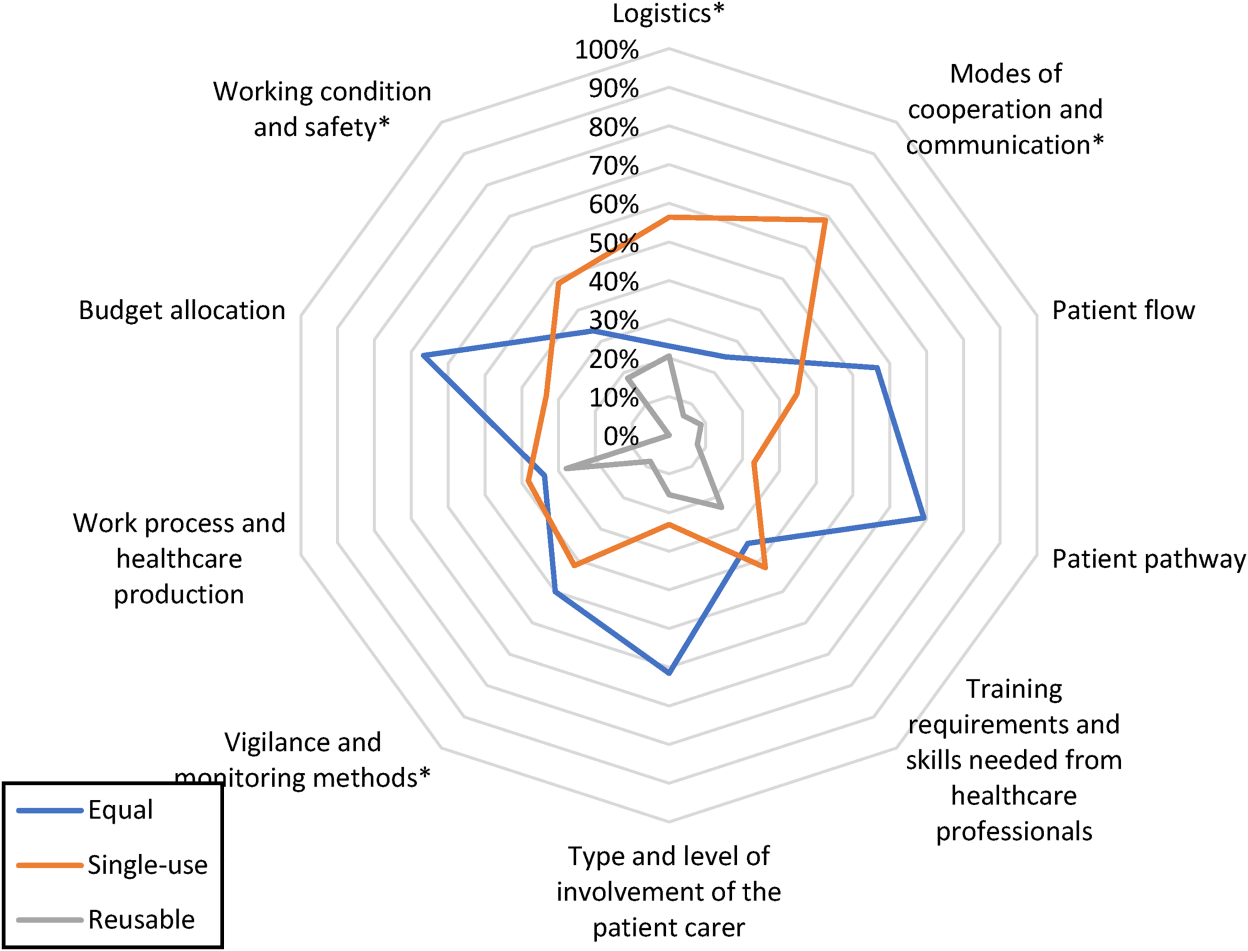
Respondents’ perceptions of organizational impact advantages. Illustration of the percentage of responses favoring single-use and reusable setups in each investigated organizational category. Responses indicating equal advantages are also included. *indicates statistical significance at α-level 0.05.

The OI preferences of the users and management are presented in Figure 5 and Table 7. The scores for SURLs were notably higher in the domain of wear and tear; 62% of doctors and 100% of managerial staff preferred SURLs. The scores for the following items clearly favored SURLs over RRLs: recording and saving of images and videos (67%), need for training of health care staff for rhinolaryngoscope handling (90%), need for communication between doctors and other staff (77%), monitoring for high hazard and infectious patients (77%), reprocessing activity (90%), and maintenance and repairs (100%) (Table 7). A statistically significant difference was observed between four OI parameters related to the perceived advantages of SURLs; these included logistics (*p* = 0.0106), modes of cooperation and communication (*p* = 0.0039), vigilance and monitoring methods (*p* = 0.0047), and working conditions and safety (*p* = 0.033). As compared to SURLs, RRLs were reported to have clear disadvantages for all items within the parameters of working conditions and safety (criteria; 8A, 8B, and 8C) and environment of healthcare staff (Table 1).

**Table 7:** Organizational impact assessment.

Organizational impact parameter		Criteria			Preferred setup		
		No.	Prof.		SURLS	Equal	RRLS
1	Work process or healthcare production (*n* = 68)	1A	d	Wear and tear problems (*n* = 13)	62%	38%	0%
		1B	d	Reliable bending (*n* = 13)	8%	46%	46%
		1C	d	Reliable image quality (*n* = 13)	15%	46%	38%
		1D	d	Training and education (*n* = 13)	29%	21%	50%
		1E	d	Record and save images videos (*n* = 14)	67%	25%	8%
		1F	m	Wear and tear problems (*n* = 3)	100%	0%	0%
Percentage of burden statements					38%	34%	28%
2	Patient pathways (*n* = 13)	2A	d	Need of transport of RL or patient (*n* = 13)	23%	69%	8%
Percentage of burden statements					23%	69%	8%
3	Patient flow (*n* = 23)	3A	d	Waiting time (*n* = 13)	23%	69%	8%
		3B	n	Availability of RL (*n* = 3)	50%	40%	10%
Percentage of burden statements					35%	57%	9%
4	Type and level of involvement of the patient/caregiver (*n* = 13)	4A	d	Involvement of patients or caregivers (*n* = 13)	23%	62%	15%
Percentage of burden statements					23%	62%	15%
5	Training requirement and skills needed from healthcare professionals (*n* = 23)	5A	d	Training requirements of doctors to handle and use the RL (*n* = 13)	15%	54%	31%
		5B	n	Training requirements of HCSs to handle the RL (*n* = 10)	90%	0%	10%
		5C	m	Skills needed from executives to manage their stocks (*n* = 3)	0%	67%	33%
Percentage of burden statements					42%	35%	23%
6	Modes of cooperation and communication (*n* = 16)	6A	d	Between doctors and colleagues (*n* = 13)	77%	23%	0%
		6B	m	Between management and the department (*n* = 3)	33%	33%	33%
Percentage of burden statements					69%	25%	6%
7	Vigilance and monitoring methods^a^ (*n* = 36)	7A	d	Monitoring of high hazard or infectious diseases (*n* = 13)	77%	8%	15%
		7B	d	Incident monitoring (*n* = 13)	0%	100%	0%
		7C	n	Administrative work (*n* = 10)	50%	40%	10%
Percentage of burden statements					42%	50	8%
8	Working conditions and safety^a^ (*n* = 33)	8A	d	Exposure to infectious agents (*n* = 13)	46%	54%	0%
		8B	n	Exposure to infectious agents (*n* = 10)	50%	20%	30%
		8C	n	Exposure to chemicals (*n* = 10)	50%	20%	30%
Percentage of burden statements					48%	33%	18%
9	Accessibility	–	–	Not applicable	NA	NA	NA
Percentage of burden statements					–	–	–
10	Budget allocation (*n* = 6)	10A	m	Complexity of budgeting to ensure RL availability (*n* = 3)	33%	67%	0%
		10B	m	Cost transparency (*n* = 3)	33%	67%	0%
Percentage of burden statements					33%	67%	0%
11	Architectural and infrastructural design	–	–	Not applicable	NA	NA	NA
Percentage of burden statements					–	–	–
12	Logistics^a^ (*n* = 39)	12A	d	Transportation management (*n* = 13)	31%	23%	46%
		12B	m	Appropriate number of RRLs (*n* = 3)	33%	67%	0%
		12C	m	Maintenance, repair and sequestration management (*n* = 3)	100%	0%	0%
		12D	n	Reprocessing activity (*n* = 10)	90%	0%	10%
		12E	n	Preparation activity (*n* = 10)	50%	40%	10%
Percentage of burden statements					56%	23%	21%

Results of the organizational impact survey. Profession (Prof.) indicates whether the question was answered by ENT doctors (d), managerial staff (m), or other healthcare professionals involved in the process such as nurses and technicians (n). The respondents' perceived advantages for each category is calculated as the weighted average of all data points within that category.

^a^ Indicates statistically significant difference between reusable and single-use rhinolaryngoscopes.

## 4 Discussion

This study, which compared the efficiency, economic considerations, and OI of SURLs and RRLs, provides important insights into the advantages and disadvantages associated with single-use and reusable flexible rhinolaryngoscopes.

The utility of SURLS vs. RRLS showed no statistically significant differences in terms of procedure duration, image quality, or maneuverability; both RRLS and SURLs were found to be equally satisfactory in terms of these parameters. According to the doctors, the main advantages of the SURLs included the perceived ease of recording and saving of images and videos and a lesser need for communication (between doctors and other staff), which could be time saving. In addition, the staff did not require much education for handling of SURLs, as there was no need for reprocessing, cleaning, or maintenance and repairs. This offered benefits in terms of requirements for personnel and other resources; notably, these are important factors to consider in healthcare settings.

The micro-costing analysis revealed the estimated per procedure cost for RRLs to be SEK 536 (€46.87) which is low compared to previous studies. This can be partly explained by high volumes and effective cleaning processes. Other studies hva shown higher costs for RRLs. In this context, Walczak et al. demonstrated that a SURL (aScope 4 RhinoLaryngo Slim) lowered costs compared to its reusable counterparts, at $27.52 (€25.06^1^). They also found that the cost savings with the single-use setup were even more considerable on considering longer-term usage scenarios; this was attributed to longer annuitization of the capital equipment (e.g., processors). Mistry et al. found that the single-use rhinolaryngoscope, aScope 4 RhinoLaryngo Slim, reduced costs by £73 (€84.34^2^) compared to reusable video rhinolaryngoscopes (9). They also found it to be cost-equivalent compared to eyepiece rhinolaryngoscopes in the outpatient clinic.

It is worth noting that costs represent only one dimension of the multifaceted decision-making process pertaining to the adoption of SURLs. In addition, the costs for RRLs may be higher in units where fewer procedures are performed (and reprocessing is therefore not as efficient as in the present study, where the cost per procedure was lower than that seen in other comparable studies) (10).

In this study, the SURL was associated with a more favorable OI compared to the RRL; this factor requires attention during selection of flexible rhinolaryngoscopes in the clinical environment. For instance, ORL clinics where personnel are less available, the use of SURLs require less maintenance and investment in specialized reprocessors. In terms of ambulatory and emergency logistics, the ability to use a flexible rhinolaryngoscope without having to consider the risk of contamination and subsequent cleaning of the endoscope (while maintaining image quality and maneuverability) could represent an advantage.

In this context, Roussel et al. emphasized the significance of considering non-clinical factors alongside clinical and economic criteria when assessing medical devices (4). Their proposed framework was later identified as the sole published method for evaluating OI in healthcare (by Pascal et al.) (11). With the worldwide problem of healthcare staff shortages, and a reprocessing of reusable endoscopes (that requires cooperation and involves staff exposure to chemicals and infectious agents), the aspects of OI warrant consideration in the decision-making process. In a study by Châteauvieux et al., the organizational and economic factors associated with the use of single-use flexible bronchoscopes were assessed. They found that although it was considered a costly alternative in the studied institution, the single-use setup offered numerous advantages (3). In facilities similar to ours, where high volumes of laryngoscopic procedures are performed, efficient careful cleaning and handling results in a per-use cost of RRL that is at or below the cost of using a SURL platform. The advantages may be greater in bronchoscopes and laryngoscopes with a working channel as the cleaning process is more complicated and the risk for contamination is greater than in the laryngoscopes in the present study that have no working channels (12).

The challenges posed by the recent Coronavirus 2019 pandemic have underscored the need for efficient and resource-conserving practices, especially in the context of emerging staff shortages in many health care systems over the world. In this context, Cooper and Shakeel (2020) introduced a SURL in response to the restrictions imposed by the pandemic (13). Their SURL ensured patient and staff safety and also streamlined procedures. The potential cost-minimizing benefits of single-use devices in these settings become even more significant when considering the challenges associated with management of limited resources and staff.

### 4.1 Strengths and limitations

This study did not include any analyses of the environmental impact of SURLS vs. RRLS which is, of course, an important factor to consider. The manufacturing process for a flexible rhinolaryngoscope includes the need for raw materials, energy, and manufacturing facilities. However, the reusable scopes need to be analyzed from the perspective of energy requirements for cleaning and the disposal and recycling of waste water and detergents. To comprehensively evaluate environmental impact, it is essential that a life cycle assessment approach is employed; this utilizes third-party assessments and encompasses all relevant stages. Several initiatives with different methodologies have been taken to compare single-use and reusable endoscopy setups. Although the findings from several studies suggest that the environmental impact of single-use endoscopy setups is not significantly worse than that of reusable (14–17), other studies have gotten opposite results (18).

Comparing SURL and RRL, it is important to acknowledge that while the RRL C-Mac 8403 zx® from Karl Storz serves as the comparator in our study, other RRLs may yield different results. Our investigation site only used C-Mac 8403 zx® from Karl Storz. This restricted our ability to compare SURL to a wider range of RRL types directly. Additionally, the age and wear and tear of the RRLs could have impacted the rating of image quality and future studies should aim to include additional study group analysis and a diverse selection of RRLs to enhance the generalizability of the findings.

A major strength of this study is that the flexible rhinolaryngoscopes were used at a university hospital where ambulatory ORL assessments using a flexible endoscope are common. The patient volumes also ensured that the participating doctors were experienced with both studied endoscopes and the ambulatory assessment setting. The validity of the study is also supported by the fact that it was performed during every-day clinical activities, and not in a controlled research environment. A majority of the procedures were indicated due to respiratory difficulties, issues with tracheostomy, and airway bleeding; these represent the acute or subacute cases that require ambulatory ORL care in a major hospital. Finally, the study periods were randomized as well as the order of the questions in the OI questionnaires which reduced the risk of question order bias.

One potential limitation of this research paper is the bias that may arise from the study being funded by the Ambu A/S, however, the published protocol with defined aim, method, and outcome measures enhances the transparency.

The scope of our study is limited by the fact that it included a relatively small number of procedures and few users, and they were performed in a heterogeneous patient population; this could have affected the results. In addition, the participants were not blinded to the aim of the study or to the type of flexible endoscope used. However, the two types of endoscopes used were already in regular and interchangeable use at the clinic, and the examining doctors, staff, and executives were well-versed in their use. Finally, despite randomization, the study had an open design study and blinding was not possible; this was associated with a risk of bias.

While our study has provided insights into the economic and organizational aspects of sustainability between SURL and RRL, it is essential to note that we have not fully embraced the comprehensive sustainability framework advocated in existing literature (19). Given that our study focused on specific sustainability aspects, future investigations should aim to explore and incorporate all aspects of sustainability (environmental, social, clinical, and economic) to ensure a thorough evaluation of both arguments and blind spots.

## 5 Conclusion

This prospective comparative study found single-use and reusable rhinolaryngoscopes to offer similar performance, image quality, and maneuverability in a tertiary ORL ambulatory setting. In addition, the findings suggested an improved organizational impact with single-use endoscopes. The costs associated with RRLs were found to be lower in this high-volume clinic. It is essential that healthcare institutions consider these factors during decision-making for the adoption of such devices.

## Data Availability

The raw data supporting the conclusions of this article will be made available by the authors, without undue reservation.

## Footnotes

^1^Exchange rate 0.91.

^2^Exchange rate 1.16.
