# Supplementary material for: Comparison of utility and organizational impact of reusable and single-use rhinolaryngoscopes in a tertiary otorhinolaryngology department
Source: Front Surg. 2024 Oct 7;11:1380571. doi: 10.3389/fsurg.2024.1380571 (PMC11491433; doi:10.3389/fsurg.2024.1380571)
Supplement: Supplementary file 1 [file Datasheet1.pdf]

Dear Participant,

You are invited to participate in our study of the organizational impact of rhinolaryngoscopy in procedures away from the ENT clinic. Please answer the following questions on the organizational impact of single-use Ambu rhinolaryngoscopes (Ambu aScope4 RhinoLaryngo) and conventional reusable rhinolaryngoscopes used in the procedures away from the ENT clinic.

Your participation in this study is completely voluntary. However, it is very important for us to learn your opinions.

The questionnaire consists of XX questions and is estimated to take XX to XX minutes to complete. The outcome will be submitted for a suitable peer-reviewed journal.

In the questionnaire, you will be asked to choose between "Ambu single-use rhinolaryngoscope", "Neutral", or "Conventional Reusable Rhinolaryngoscope". Please avoid choosing, Neutral, when possible, as this implies that you are indifferent to the question. You will receive the questions in a random order, and you should only complete the survey once. Only complete questionnaires will be included in the final analysis.

If you have questions at any time about the survey or the procedures, you may contact XXX at XXX. Thank you very much for your time and support. Please start the survey and in the questionnaire continue to the next question by clicking on the "Next" button.

How many procedures have you performed with the single-use Ambu rhinolaryngoscope?

1. 1-5 procedures
2. 5-10 procedures
3. 11-15 procedures
4. 16-20 procedures
5. >20 procedures

Have you used reusable rhinolaryngoscope?

1. Yes
2. No **(EXCLUDE IF NO)**

(Introduction – Randomization starts from this point)
Please answer the following questions on the organizational impact of single-use Ambu rhinolaryngoscopes (Ambu aScope4 RhinoLaryngo) and conventional reusable rhinolaryngoscopes used in consult procedures. The questions and the answers will be randomized so please pay attention to each question, since the order of the answers will differ.

**Work process and healthcare production**

With which rhinolaryngoscope do you experience most wear and breakage issues?

| | Ambu single-use rhinolaryngoscope | Neutral | Conventional reusable rhinolaryngoscope |
|---|---|---|---|
| Please answer from your general point of view. | ❏ | ❏ | ❏ |

With which rhinolaryngoscope do you experience the most reliable performance regarding bending capability?

| | Ambu single-use rhinolaryngoscope | Neutral | Conventional reusable rhinolaryngoscope |
|---|---|---|---|
| Please answer from your general point of view. | ❏ | ❏ | ❏ |

With which rhinolaryngoscope do you experience the most reliable performance regarding image quality?

| | Ambu single-use rhinolaryngoscope | Neutral | Conventional reusable rhinolaryngoscope |
|---|---|---|---|
| Please answer from your general point of view. | ❑ | ❑ | ❑ |

Training and education
If you were to train a medical student or new ENT doctor in rhinolaryngoscopy, which rhinolaryngoscope would you prefer?

| | Ambu single-use rhinolaryngoscope | Neutral | Conventional reusable rhinolaryngoscope |
|---|---|---|---|
| Please answer from your general point of view. | ❑ | ❑ | ❑ |

Recording and saving images and video's
If you want to record and save images from your rhinolaryngoscopy, which rhinolaryngoscope would you prefer?

| | Ambu single-use rhinolaryngoscope | Neutral | Conventional reusable rhinolaryngoscope |
|---|---|---|---|
| Please answer from your general point of view. | ❑ | ❑ | ❑ |

**Patient pathway**
Prior to performing a rhinolaryngoscopy, when do you experience the least effort for transportation; either of the rhinolaryngoscope to the patient or of the patient to the rhinolaryngoscope?

| | Ambu single-use rhinolaryngoscope | Neutral | Conventional reusable rhinolaryngoscope |
|---|---|---|---|
| Please answer from your general point of view. | ❑ | ❑ | ❑ |

**Patient flow**
With which rhinolaryngoscope do you expect the longest time interval from indication for rhinolaryngoscopy until the start of the procedure?

| | Ambu single-use rhinolaryngoscope | Neutral | Conventional reusable rhinolaryngoscope |
|---|---|---|---|

| | | | |
|---|---|---|---|
| Please answer from your general point of view. | ❏ | ❏ | ❏ |

**When waiting for a rhinolaryngoscope to become available, how long do you typically have to wait?**

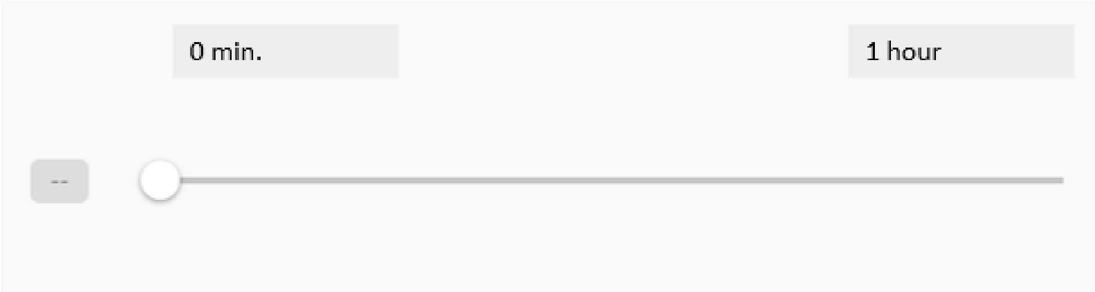


**Type and level of involvement of the patient/carer**
With which rhinolaryngoscope is it most cumbersome to involve patients or carers in the procedure and subsequent diagnosis or clinical decision? (e.g. show or present the procedure and findings during or following the procedure)

| | Ambu single-use rhinolaryngoscope | Neutral | Conventional reusable rhinolaryngoscope |
|---|---|---|---|
| Please answer from your general point of view. | ❏ | ❏ | ❏ |

**Training requirements and skills needed from healthcare professionals**
If you were a new ENT doctor, which rhinolaryngoscope would demand least training to operate?

| | Ambu single-use rhinolaryngoscope | Neutral | Conventional reusable rhinolaryngoscope |
|---|---|---|---|
| Please answer from your general point of view. | ❏ | ❏ | ❏ |

**Modes of cooperation and communication**
Which rhinolaryngoscope requires most coordination with colleagues to ensure ready-to-use rhinolaryngoscopes?

| | Ambu single-use rhinolaryngoscope | Neutral | Conventional reusable rhinolaryngoscope |
|---|---|---|---|
| Please answer from your general point of view. | ❏ | ❏ | ❏ |

**Vigilance and monitoring methods**
Which rhinolaryngoscope requires the least vigilance and monitoring of high hazard or highly infectious diseases? E.g. Creuzfeldt-Jakob disease or COVID-19.

| | Ambu single- | Neutral | Conventional |
|---|---|---|---|

| | use rhinolaryngoscope | | reusable rhinolaryngoscope |
|---|---|---|---|
| Please answer from your general point of view. | ❑ | ❑ | ❑ |

If you experience a device malfunction during a rhinolaryngoscopy, with which rhinolaryngoscope do you most easily report the incidence to relevant patient safety authorities?

| | Ambu single-use rhinolaryngoscope | Neutral | Conventional reusable rhinolaryngoscope |
|---|---|---|---|
| Please answer from your general point of view. | ❑ | ❑ | ❑ |

**Working conditions and safety**

With which rhinolaryngoscope do you feel most exposed to infectious agents?

| | Ambu single-use rhinolaryngoscope | Neutral | Conventional reusable rhinolaryngoscope |
|---|---|---|---|
| Please answer from your general point of view. | ❑ | ❑ | ❑ |

**Logistics**

When performing a rhinolaryngoscopy, with which rhinolaryngoscope do you experience the least logistical burden associated with transportation within and between departments?

| | Ambu single-use rhinolaryngoscope | Neutral | Conventional reusable rhinolaryngoscope |
|---|---|---|---|
| Please answer from your general point of view. | ❑ | ❑ | ❑ |

Please, comment on other organizational aspects that might affect your workflow that were not discussed in this questionnaire.

| |
|---|
| |

Dear Participant,

You are invited to participate in our study of the organizational impact of rhinolaryngoscopy in procedures away from the ENT clinic. You are requested to answer the questions from a managerial perspective. Please answer the following questions on the organizational impact of single-use Ambu rhinolaryngoscopes (Ambu aScope4 RhinoLaryngo) and conventional reusable rhinolaryngoscopes used in the procedures away from the ENT clinic. Your participation in this study is completely voluntary. However, it is very important for us to learn your opinions.

The questionnaire consists of XX questions and is estimated to take XX to XX minutes to complete. The outcome will be submitted for a suitable peer-reviewed journal. In the questionnaire, you will be asked to choose between "Ambu single-use rhinolaryngoscope", "Neutral", or "Conventional Reusable Rhinolaryngoscope". Please avoid choosing, Neutral, when possible, as this implies that you are indifferent to the question.

You will receive the questions in a random order, and you should only complete the survey once. Only complete questionnaires will be included in the final analysis.

If you have questions at any time about the survey or the procedures, you may contact XXX at XXX

Thank you very much for your time and support.

Please start the survey and in the questionnaire continue to the next question by clicking on the "Next" button.

(Introduction – Randomization starts from this point)
Please answer the following questions on the organizational impact of single-use Ambu rhinolaryngoscopes (Ambu aScope4 RhinoLaryngo) and conventional reusable rhinolaryngoscopes used in consult procedures. The questions and the answers will be randomized so please pay attention to each question.

**Work process and healthcare production**
With which rhinolaryngoscope do you experience the least wear and breakage issues?

| | Ambu single-use rhinolaryngoscope | Neutral | Conventional reusable rhinolaryngoscope |
|---|---|---|---|
| Please answer from your general point of view | ❑ | ❑ | ❑ |

**Training requirements and skills needed from healthcare professionals**
With which rhinolaryngoscope do you need the highest level of training to manage the stock of endoscopes and endoscope-related equipment?

| | Ambu single-use rhinolaryngoscope | Neutral | Conventional reusable rhinolaryngoscope |
|---|---|---|---|
| Please answer from your general point of view | ❑ | ❑ | ❑ |

**Modes of cooperation and communication**
Which rhinolaryngoscope requires the least amount of cooperation and communication within your department and with other departments? (E.g., procurement, engineering, reprocessing unit, or infection control)

| | Ambu single-use rhinolaryngoscope | Neutral | Conventional reusable rhinolaryngoscope |
|---|---|---|---|
| Please answer from your general point of view | ❑ | ❑ | ❑ |

**Budget allocation**
Which rhinolaryngoscope requires the least complex budgeting to ensure patient-ready endoscopes and associated technologies? Please consider investment, repair, reprocessing, facilities, etc.

| | Ambu single-use rhinolaryngoscope | Neutral | Conventional reusable rhinolaryngoscope |
|---|---|---|---|
| Please answer from your general point of view | ❑ | ❑ | ❑ |

Which rhinolaryngoscope setup has the most transparent costs?

| | Ambu single-use rhinolaryngoscope | Neutral | Conventional reusable rhinolaryngoscope |
|---|---|---|---|
| Please answer from your general point of view | ❑ | ❑ | ❑ |

**Logistics**
With which rhinolaryngoscope do you experience the smallest logistical burden associated with having too few or a surplus of rhinolaryngoscopes?

| | Ambu single-use rhinolaryngoscope | Neutral | Conventional reusable rhinolaryngoscope |
|---|---|---|---|
| Please answer from your general point of view | ❑ | ❑ | ❑ |

With which rhinolaryngoscope do you experience the biggest logistical burden associated with maintenance, repair and sequestration management?

| | Ambu single-use rhinolaryngoscope | Neutral | Conventional reusable rhinolaryngoscope |
|---|---|---|---|
| Please answer from your general point of view | ❑ | ❑ | ❑ |

Please, comment on other organizational aspects that might affect your workflow that were not discussed in this questionnaire.

| |
|---|
| |

Dear Participant,

You are invited to participate in our study of the organizational impact of rhinolaryngoscopy in procedures away from the ENT clinic. Please answer the following questions on the organizational impact of single-use Ambu rhinolaryngoscopes (Ambu aScope4 RhinoLaryngo) and conventional reusable rhinolaryngoscopes used in the procedures away from the ENT clinic.

Your participation in this study is completely voluntary. However, it is very important for us to learn your opinions.

The questionnaire consists of XX questions and is estimated to take XX to XX minutes to complete. The outcome will be submitted for a suitable peer-reviewed journal.

In the questionnaire, you will be asked to choose between "Ambu single-use rhinolaryngoscope", "Neutral", or "Conventional Reusable Rhinolaryngoscope". Please avoid choosing, Neutral, when possible, as this implies that you are indifferent to the question. You will receive the questions in a random order, and you should only complete the survey once. Only complete questionnaires will be included in the final analysis.

If you have questions at any time about the survey or the procedures, you may contact XXX at XXX. Thank you very much for your time and support. Please start the survey and in the questionnaire continue to the next question by clicking on the "Next" button.

How many procedures have you assisted with the single-use Ambu rhinolaryngoscope?

6. 1-5 procedures
7. 5-10 procedures
8. 11-15 procedures
9. 16-20 procedures
10. >20 procedures

Have you used reusable rhinolaryngoscope?

3. Yes
4. No

(Introduction – Randomization starts from this point)
Please answer the following questions on the organizational impact of single-use Ambu rhinolaryngoscopes (Ambu aScope4 RhinoLaryngo) and conventional reusable rhinolaryngoscopes used in consult procedures. The questions and the answers will be randomized so please pay attention to each question, since the order of the answers will differ.

**Training requirements and skills needed from healthcare professionals**
If you were new to rhinolaryngoscopy, which rhinolaryngoscope would require least training before you successfully, pre- and post-procedure, could set up the endoscopy equipment, clean, transport, store, and/or dispose of the rhinolaryngoscope?

| | Ambu single-use rhinolaryngoscope | Neutral | Conventional reusable rhinolaryngoscope |
|---|---|---|---|
| Please answer from your general point of view | ❑ | ❑ | ❑ |

**Logistics**
Which rhinolaryngoscope requires least time to clean, transport, store, and/or dispose?

| | Ambu single-use rhinolaryngoscope | Neutral | Conventional reusable rhinolaryngoscope |
|---|---|---|---|
| Please answer from your general point of view | ❑ | ❑ | ❑ |

When do you spend most time setting up the endoscope-associated technologies (e.g. the monitor) for a rhinolaryngoscopy?

| | Ambu single-use rhinolaryngoscope | Neutral | Conventional reusable rhinolaryngoscope |
|---|---|---|---|
| Please answer from your general point of view | ❑ | ❑ | ❑ |

**Work conditions and safety**

When transporting, cleaning, storing and/or disposing; with which rhinolaryngoscope do you feel most exposed to infectious agents?

| | Ambu single-use rhinolaryngoscope | Neutral | Conventional reusable rhinolaryngoscope |
|---|---|---|---|
| Please answer from your general point of view | ❑ | ❑ | ❑ |

When transporting, cleaning, storing and/or disposing; with which rhinolaryngoscope do you feel least exposed to chemicals?

| | Ambu single-use rhinolaryngoscope | Neutral | Conventional reusable rhinolaryngoscope |
|---|---|---|---|
| Please answer from your general point of view | ❑ | ❑ | ❑ |

**Vigilance and monitoring methods**

With which rhinolaryngoscope do you spend least time on administrative work? (E.g., paperwork associated with microbiological sampling, endoscope tracking etc.)

| | Ambu single-use rhinolaryngoscope | Neutral | Conventional reusable rhinolaryngoscope |
|---|---|---|---|
| Please answer from your general point of view | ❑ | ❑ | ❑ |

**Patient flow**

Which rhinolaryngoscope do you experience to be most frequently unavailable pre-procedure?

| | Ambu single-use rhinolaryngoscope | Neutral | Conventional reusable rhinolaryngoscope |
|---|---|---|---|
| Please answer from your general point of view | ❑ | ❑ | ❑ |

Please, comment on other organizational aspects affected by single-use vs. reusable rhinolaryngoscopes.
